# Effects of a Nomadic Lifestyle, Gender, and Education on Attitudes of Chinese People Towards Animal Welfare

**DOI:** 10.3390/ani16081194

**Published:** 2026-04-14

**Authors:** Xintong Li, Xiao Jin, Xuan Gu, Zhipeng Han, Clive J. C. Phillips

**Affiliations:** 1 College of Animal Science, Inner Mongolia Agricultural University, Zhaowuda Road 306, Hohhot 010018, China; lixintongaa@163.com (X.L.); hzp4639@163.com (Z.H.); 2 Department of Social Work, School of Philosophy and Social Development, Shandong University, Jinan 250100, China; guxuan@sdu.edu.cn; 3 Centre for Animal Protection Studies, Shandong University, Jinan 250100, China; 4 Sustainability Policy (CUSP) Institute, Curtin University, Kent St., Bentley, Perth 6102, Australia; clive.phillips@curtin.edu.au; 5 Institute of Veterinary Medicine and Animal Science, Estonia University of Life Sciences, Kreutzwaldi 1, 51014 Tartu, Estonia

**Keywords:** animal welfare, nomadism, education level, gender, China

## Abstract

Attitudes towards animal welfare depend on people’s lifestyles and education levels, yet little is known about the attitudes of nomadic people. We distributed a questionnaire and collected 1660 valid responses, representing individuals with or without nomadic connections and varying education levels and genders. We used ordinal logistic regression to analyze the impact of these two factors on perceptions, attitudes, and behavioral intentions related to animal welfare. Women and those with higher education levels tended to hold more positive attitudes toward animal welfare, but for farm animals, this was only extended to common female reproducing animals, such as cows, sheep, goats, and laying hens, but not other farm animals, such as beef cattle and broiler chickens. This may reflect a greater sensitivity towards reproducing animals. The influence of nomadic connections on attitudes towards animal welfare was not linear—respondents with nomadic connections themselves or family members of the same generation generally had more negative attitudes, while those with grandparents with nomadic connections had more positive attitudes on several animal welfare issues. This may reflect a generational change in the attitudes of nomadic people towards animals.

## Simple Summary

In China, animal welfare is gradually becoming more recognized. A survey activity on public attitudes towards animal welfare which obtained 1660 valid responses was conducted to analyze how three factors, the nomadic connections of respondents, gender, and their education levels, influence their perception of, and agreement with, animal welfare. Women and those with higher education levels held more positive attitudes towards animal welfare, and in the case of farm animals, women generally had more positive attitudes towards female animals kept for breeding, such as cows, sheep, and laying hens, but not beef cattle or broiler chickens that are raised to be slaughtered for meat. Those with lower education levels showed stronger resistance to invasive rearing management methods. The influence of nomadic connections on attitudes towards animal welfare was complex—if they or their current-generation family members were nomadic, they tended to hold more negative attitudes towards animals, whereas respondents with grandparents who had a nomadic connection tended to have more positive attitudes towards animal welfare issues. This may reflect a generational change in attitudes of nomadic people towards animals.

## 1. Introduction

Nomadism is a way of life in which people move regularly with their herds in search of pasture and water, and this migratory life is dispersed in time and space [1]. However, in today’s rapidly developing globalization, influenced by industrialization, urbanization, and modern national governance systems, nomadic life is gradually declining globally [2]. With the degradation of grasslands and the need for a more sustainable system, herders around the world have over the past two decades transitioned from a nomadic lifestyle to a settled one [3,4]. At the same time, nomadic culture faces the risks of fragmentation and even disappearance. Multiple countries have nominated nomadic-related crafts for the UNESCO Intangible Cultural Heritage List and their respective national intangible cultural heritage protection lists [5,6,7,8].

When lifestyles change so drastically, public perception of different dimensions of animal welfare, such as nutrition, environment, health and behavior, may also shift. This cognitive difference is crucial for adapting animal welfare measures that align with local cultural perceptions under modern management systems. In animal welfare, the conceptualization of what constitutes good welfare has evolved from the Five Freedoms—which focused on the avoidance of negative states such as hunger, thirst, and distress—to more comprehensive frameworks like the Five Domains Model [9,10]. The Five Domains model emphasizes the necessity of pursuing positive welfare states across nutrition, environment, health, and behavioral interactions. Although these frameworks are widely accepted in the scientific community, there is still a lack of public understanding of them. However, it remains unclear whether individuals from different life backgrounds and social identities exhibit differences in their recognition of these refined dimensions of welfare, and how such differences are manifested. Through a large-scale survey of the public, the present study includes gender, education level, and nomadic connections as core influencers of animal welfare analysis in northern China.

Nomadism often limits access to education, which, as a core indicator of a respondent’s cognitive ability, knowledge base, and socialization, has always played the role of an ethical concept shaper in modern society [11]. Although there is little information about how education influences attitudes towards animal welfare in people with connections to nomadism, a study among farm workers on 31 large-scale dairy farms in Midlands Province, Zimbabwe, found that 67% of farm workers were poorly informed about animal welfare, and their depth of understanding was significantly correlated with their education levels [1]. Education may include didactic teaching about animal welfare. For example, an online survey of animal science students found that the majority of students strongly agreed that an animal welfare course was an important part of the animal science curriculum [3,12]. The influence of education level on the attitudes of people with connection to a nomadic way of life towards animal welfare is worth exploring in depth. Therefore, the present survey includes education level as one of the core influence factors, aiming to systematically analyze the differences in respondents’ perceptions and attitudinal tendencies towards animal welfare under education levels [12].

Gender is one of the demographic variables widely studied in various survey-based literature, and some international surveys have already addressed the impact of gender on animal welfare [13,14]. In the context of China’s agricultural transformation, consumers are rapidly shifting their focus from survival needs to quality attributes, making it crucial to understand the specific responses of Chinese female consumers to local animal welfare-related issues. With China’s rapid modernization, women’s roles in society have undergone significant changes, particularly reflected in their influence on family consumption and lifestyle choices. Therefore, it is particularly important to examine whether the global trend that women focus on animal welfare more than men still holds true in China.

The impact of culture on perceptions of animal welfare arises from the long-term interplay between humans and their environments and is defined by culturally distinctive ways. In the traditional culture of nomadism, animals are not only food resources for survival but also carriers of social structure, spiritual support, and ecological cognition. Surveys conducted in Ethiopia have shown that traditional lifestyles such as farming and nomadism have impacted people’s attitudes towards animal welfare [4]. Due to local socio-cultural, demographic, and agroecological factors, there are differences in the attitudes and practices of rural households towards animal welfare, and people’s perceptions and practices of animal welfare are profoundly affected by their lifestyles [4]. In the Sami community in Norway, traditional reindeer herding, fishing, and hunting are an important part of their lives. These traditional activities are related to their animal welfare attitudes [15]. In northern China, the nomadic culture in the Mongolian Plateau regards the horse as the “soul of the steppe”, and the respect for animals pervades their daily care and migration rituals [16]. The rice-farming societies of the Yangtze River Basin in central China developed a simple ethic, with the ox as the centerpiece of cultivation, being accorded a status close to that of a family member [5]. These indigenous patterns of interaction, which are both interdependent and intimate, have led traditional communities to spontaneously develop practices for seasonal rotational grazing systems to avoid the degradation of both their flocks/herds and the pastures [6]. The Qimin Yaoshu (Essential Techniques for the Welfare of the People), a book on agriculture and animal husbandry in ancient China compiled by Jia Sixie in the 6th century CE, provides some of the earliest systematic guidelines for managing draft animals such as cattle, horses, donkeys, and mules. It recommends feeding three times daily to support rumination, watering at three intervals daily to prevent overexertion on an empty stomach, and in winter, trimming the animals’ coats and covering them with felt to protect against frostbite. These instructions reflect an early recognition of the need to balance agricultural productivity with the health and welfare of working animals in pre-modern China [7]. Although these practices are not labeled as modern concepts of “animal welfare,” they are inherently linked to the ethical principles of reducing suffering and safeguarding natural behaviors whilst reflecting traditional culture’s intuitive understanding of the symbiosis of life.

However, the existing animal welfare evaluation systems are predominantly based on Western urban civilization. Most existing literature either treats cultural background in broad terms or focuses on general demographic characteristics, leaving the specific influence of nomadic background unexplored [17]. Nomadic backgrounds, which represent long-term, experience-based human–animal interactions, have rarely been incorporated as independent explanatory variables in empirical studies. Moreover, the mechanisms through which such backgrounds shape different dimensions of animal welfare attitudes remain unclear. In China, there is a lack of studies that incorporate nomadic lifestyle into animal welfare, and how nomadism associates with the attitudes towards animal welfare is unclear [18,19].

It is still unclear in previous studies whether the public, with different culture backgrounds and different identities, exhibits differences in recognizing the refined dimensions of animal welfare, and in what manners such differences manifest. Through a large-scale survey of the public, the present study includes gender, education level, and nomadic connections as the core dimensions of analysis. This study aims to examine the independent effects of nomadic background, gender, and education level on the perception of and attitudes towards animal welfare and further compare their different roles across various dimensions of welfare. Ultimately, within the Chinese context, it seeks to provide a basis for incorporating variables such as nomadic background into the analytical framework for animal welfare.

## 2. Materials and Methods

### 2.1. Survey Methodology

This study used a questionnaire survey distributed in July 2023, aiming to understand public attitudes towards animal welfare in a region of northern China with significant nomadic connections and the influence factors. The questionnaire design was based on animal welfare attitude issues and appropriately adjusted according to the cultural background in Inner Mongolia to ensure cultural appropriateness and respondents’ comprehension. For instance, local livestock were added to the species list. The survey was originally drafted and distributed in standard Mandarin (written in Simplified Chinese). Due to the national standardized compulsory education system, the vast majority of the regional population—including both the Han majority and the ethnic Mongolian population who are largely bilingual—were fully proficient in reading and writing standard Mandarin.

Considering the extensive and dispersed geographical distribution of pastoral residents, this study utilized online questionnaires disseminated through electronic survey platforms, including Wenjuanxing, WeChat, Xiaohongshu, Weibo, and other public social media platforms. We recruited university student volunteers to forward the survey to their family members, relatives, and acquaintance networks, and then had them further disseminate it within their communities, with the aim of enhancing sample coverage and diversity. The questionnaire began with a brief introduction, informing respondents that this study is solely for academic purposes, participation is entirely voluntary, and responses are anonymous.

The questionnaire (see Appendix A) comprised three main sections. The first part (Q1–7) collected demographic information, including respondents’ gender, age, ethnicity, education level, occupation, place of residence, and family connections. The second part (Q8–19) investigated respondents’ knowledge related to animal welfare, including understanding of animal welfare concepts, frequency of and reasons for animal interactions, and views on animal status and importance. The third part (Q20–25) explored respondents’ practical support and social participation, their attitudes towards animal welfare (e.g., sources of awareness, the necessity of promoting animal welfare, and responsibilities for its implementation), and whether they would be willing to pay a premium for animal welfare products and the acceptable price range.

In this study, the degree of respondents’ association with nomadic culture was measured through one question in the questionnaire: “Please indicate whether anyone in your family lives or lived a nomadic life”, with the options: great-grandparents or earlier, grandparents, parents, self or same-generation relatives, and no-one. If this question on nomadic lifestyle was answered in a positive way, it was considered that the respondent had a nomadic connection, and if “no-one” was selected, it was considered that the respondent had no nomadic connections. Education level was measured with the options: elementary school or below, junior high school, high school, technical school, university, and postgraduate. (The Chinese education system is divided into three stages: basic education, including elementary school or below and junior high school; secondary education, including technical schools and high school; and higher education, including university, postgraduate, and above.) Education level was coded as an ordinal multi-category variable.

Respondents’ attitudes were rated using a 5-point Likert scale. All response data were converted to a numeric scale of 1 to 5 for subsequent analysis, where “completely disagree” corresponds to 1 and “strongly agree” corresponds to 5, or “completely unimportant” corresponds to 1 and “very important” corresponds to 5. This means that the higher the value, the more positive the attitude, and conversely, a lower value means a more negative attitude. To ensure internal consistency, certain items were reverse-coded prior to analysis, but in our reporting, for all items, a higher score represents a more positive attitude toward animal welfare.

During the survey, a total of 1660 questionnaires were finished. The sample covered residents with different genders, ages, education levels, and occupation. The questionnaire was translated from Chinese to English for reporting purpose by ChatGPT3.5 and checked by the first and second authors.

### 2.2. Statistical Analysis

All survey data were organized and imported into IBM SPSS Statistics 27.0.1 software for analysis. First, descriptive statistics were conducted to present the demographics characteristics of the sample (including gender, age, ethnicity, education level, occupation, and type of residence). Before formal modeling, the 5-point Likert scale data were reassigned so that higher scores always indicated more positive attitudes toward animal welfare, and independent variables were converted into dummy variables for final data analyses. Subsequently, ordinal logistic regression models were used to examine the effects of education level, gender, and nomadic cultural connections on 6 questions related to animal welfare attitudes. Respondents’ degrees of agreement on animal welfare were measured using a 5-point Likert scale (1 = strongly disagree, 5 = strongly agree). Each question on animal welfare was treated as an ordinal dependent variable, with education level (elementary school or below, junior high school, high school, technical school, university, and postgraduate), lifestyle (no nomadic connections, self or relatives of same generation, grandparents, and great-grandparents or earlier), and gender as main predictor variables.

We evaluated the independence of these key variables by a Spearman’s Rank Correlation matrix. As shown in Table 1, we found that these variables were independent (gender and education, rho = 0.03, *p* = 0.22; nomadic lifestyle and education, rho = 0.033, *p* = 0.181), except for gender and nomadic lifestyle (rho = −0.061, *p* = 0.013). However, the correlation coefficient (rho) between gender and nomadic lifestyle was low; therefore, we considered the variables sufficiently independent for following analysis. Furthermore, Spearman’s rank correlation was performed to examine the relationship between region and nomadic connection. A significant correlation was observed (*p* = 0.001, see Table 2). However, to isolate the unique culture intergenerational transmission contribution of nomadic connection, nomadic connectivity was maintained as a core independent variable in subsequent multivariate analyses. During the modeling process, “university” and “no nomadic connections” were set as reference groups, because setting the group with largest number of participants as the reference makes parameter interpretation more intuitive and enhances the gap in impact degree among levels in variables. The tabulated results provide mean scores, OR values, Confidence Intervals, and *p* values have undergone the parallel line assumption.

## 3. Results

A total of 1660 valid questionnaires were collected in this study and all questions were answered. The mean completion time was approximately 270 s. There were slightly more male than female respondents and 5% of respondents did not disclose their gender (Table 3). The 18–24-year-old age group was most common (46.1%), followed 25–34 years old (21.8%), and then 35–44 years old (13.9%). In terms of ethnic identity, 54.3% of the respondents self-identified as Han Chinese, 35.6% self-identified as Mongolians, and the rest self-identified as ethnic minorities such as Hui (3.0%), Tibetan (1.5%), and Uyghur (1.4%). In response to the question about nomadic lifestyle, 524 reported that they had no nomadic lifestyle; in contrast, 210 reported themselves or their family members of the same generation had a nomadic lifestyle, 367 reported their parents had, 300 reported their grandparents had, and 259 reported their great-grandparents or earlier had. More than half of the respondents had a university degree (54.9%), 7.9% were postgraduate, and the proportion of elementary high school education or below was relatively low (4.5%). The occupational structure was relatively diverse, distributed between animal husbandry (14.0%), agriculture (11.9%), education (10.5%), and other fields. Urban residents accounted for most respondents (40.4%), followed by rural areas (30.0%) and towns (26.4%). The remaining results are divided into four parts: respondents’ views on animal welfare-related issues, attitudes toward different animals, attitudes toward animal management and care, and motivations for caring about animals. They are presented in tables containing reference data: OR values, Confidence Intervals, and *p* values.

### 3.1. Respondents’ Views on Animal Welfare-Related Issues

Table 4 shows the results of the six-point Likert scale questions on animal welfare-related issues. About two thirds of the respondents indicated that they were very familiar with or familiar with the concept animal welfare. The proportion of respondents who had not encountered the concept at all was low, approximately 14%. In terms of their interaction with animals, 43.8% of the respondents thought that the relationship was “very harmonious”, 31% thought it was “acceptable”, and about 10.0% of the respondents expressed negative emotions towards this relationship. Regarding the importance of treating animals kindly, more than half of the respondents (52.4%) thought it was “very necessary” and another 23.4% thought it was “necessary”. Correspondingly, 75.6% of the respondents thought promoting humane treatment was necessary or very necessary, and only about 10.3% held negative opinions. In terms of the relationship between animal welfare and consumer behavior, 72.8% of respondents expressed their willingness to pay a higher price for animal welfare products. About 27.2% of respondents were willing to pay 5% to 10% more, 27% were willing to pay 20%, and a small number of people were willing to pay a higher proportion, even more than 100% (7.0%).

### 3.2. Attitudes Towards Different Animals

As shown in Table 5, we examined respondents’ attitudes toward the humane treatment of different types of animals, including pets, abandoned pets, laboratory animals, farm animals, wildlife, as well as specific species within the farm animal category. Table 6 presents the results of regression analysis. Generally speaking, gender and education level were the two most significant predictors of attitude.

Regarding gender differences, women scored significantly higher than men on attitudes toward all animal groups. However, within the farm animal species, attitudes towards cows, sheep, goats, laying hens, chickens, and pigs were more benign in female than male participants, but for beef cattle, broiler chickens, camels, horses, and donkeys, there was no significant difference between male and female respondents.

Regarding education level, the lower the education level, the less willing the respondents were to believe that animals should be treated kindly. Compared with respondents with a university degree, those with primary school education or below had significantly lower odds of supporting humane treatment across all animal categories. For example, the odds that respondents with primary education or below supported kind treatment of pets were only 35% of that of the university group (OR = 0.35, *p* < 0.001), and this proportion further decreased to 25% regarding attitudes towards farm animals (OR = 0.25, *p* < 0.001). Similar trends were observed in the junior high school and high school education groups; although their OR values were slightly higher than those of the primary school group, they were still significantly lower than those of the university group. For example, high school graduates were less likely to support humane treatment of wildlife than university graduates (OR = 0.55, *p* < 0.001). When it came to farm animal species, high school graduates were only 60% (OR = 0.60, *p* < 0.01) and 50% (OR = 0.50, *p* < 0.001) as likely as their university graduates to support kind treatment of wildlife.

Considering nomadic connections, compared with those without any nomadic connections, differences were generally confined to those who themselves or relatives of the same generation were of nomadic connections, not their grandparents or earlier. These respondents believed less than those of no nomadic connections that pets, abandoned pets, and laboratory animals should be treated kindly. In the case of pets, this extended to those whose parents had nomadic connections. There was no effect of nomadic connections on beliefs that wildlife or farm animals should be treated kindly. Considering specific farm animal species, those who themselves or relatives of the same generation were of nomadic connections had less concern for treating broiler chickens, pigs, sheep, and camels kindly, but not cows or beef cattle. Overall, while gender and education demonstrated broad and consistent effects across animal categories, the impact of nomadic background was more specific, primarily affecting attitudes toward companion animals and selected livestock species rather than wildlife or farm animals as a general category.

### 3.3. Attitudes Towards the Management and Care of Animals

As shown in Table 6, the next part of the questionnaire was about respondents’ views on the importance of various aspects of farm animal welfare. Female respondents were more concerned with disease and injury, enough space, diet, drinking water, pain, comfort and absence of fear affected animal welfare than male respondents, but there were no gender differences in beliefs about the importance of a natural environment, physical fitness, humane management, and expression of natural instincts. Similarly, females did not consider procedures such as ear tagging, castration, and tail docking less acceptable than males, and neither did they think farm animals should be provided with a comfortable and pleasant living environment or that animals should be completely dead before being cooked more than males. However, they were stronger supporters of permits for animal care on farms, animal protection legislation and organizations, and avoidance of suffering, although the direction of this effect should be interpreted cautiously in light of item wording. Overall, women demonstrated stronger concern for welfare-related living conditions, regulatory oversight, and ethical consumption.

The impact of education level on welfare awareness was similar to that mentioned earlier, with the groups with lower education levels demonstrating significantly weaker welfare awareness in the majority of questions (OR < 1, *p* < 0.05). The smallest ORs were for those respondents educated to elementary school, technical school or below, with those educated to high school having more awareness of effects on welfare but still less than that of university graduates. Postgraduates had similar views to those of university graduates. In contrast, postgraduate respondents did not differ significantly from university graduates on most items, indicating that the principal divide lies between tertiary and non-tertiary education levels. However, there was no education effect on the acceptability of animal procedures.

Welfare awareness differed between respondents who themselves or their relatives of the same generation had nomadic connections, compared with those with no nomadic connections. The former had less awareness of relation to all aspects of animal welfare than the latter, with the exception of acceptability of animal procedures, including natural environment, physical fitness, diseases and injuries, enough space, humane management, natural behavioral expression, comfortable environment, absence of fear, and drinking water quality. There were some instances of grandparents with nomadic connections having greater awareness than those without nomadic connections in relation to pain, fear, drinking water quality, and acceptability of animal procedures. Not purchasing animal products if the animals suffered was supported more by respondents whose grandparents or great grandparents had nomadic connections.

In summary, attitudes towards animal management and care were strongly associated with gender and education level, with women and more highly educated respondents expressing greater welfare awareness. The influence of nomadic background was generationally differentiated: immediate nomadic background was generally associated with lower welfare awareness, whereas more distant nomadic ancestry was linked to heightened concern in selected domains.

**Table 6:** Significant effects of gender, education and nomadic relations on respondents’ attitudes towards specific aspects of animal welfare.

Part 2						
**Q3: To What Extent Do You Think the Following Factors Affect the Welfare of Farm Animals?**						
**Variable**	**OR**	**95% CI**	***p*** **-Value**	**OR**	**95% CI**	***p*** **-Value**
	**Natural environment**			**Physical fitness**		
Gender						
Female	1.11	0.92–1.34	0.272	1.11	0.92–1.34	0.289
Undisclosed	1.00	0.66–1.51	0.997	0.70	0.46–1.05	0.088
Male	Reference category					
Education						
Elementary school or below	0.37	0.24–0.57	0.000	0.29	0.19–0.44	0.000
Junior high school	0.54	0.38–0.77	0.001	0.42	0.30–0.60	0.000
High school	0.63	0.49–0.82	0.001	0.70	0.54–0.91	0.008
Technical school	0.43	0.32–0.58	0.000	0.28	0.20–0.38	0.000
Postgraduate	0.82	0.58–1.15	0.255	0.97	0.68–1.38	0.852
University	Reference category					
Nomadic relations						
Great-grandparents or earlier	1.170	0.89–1.55	0.268	0.949	0.71–1.26	0.717
Grandparents	0.946	0.73–1.23	0.682	1.151	0.87–1.52	0.319
Parents	1.176	0.92–1.51	0.203	0.911	0.71–1.18	0.474
Self or relatives of the same generation	0.711	0.53–0.96	0.024	0.619	0.46–0.84	0.002
No nomadic connections	Reference category					
	**Diseases and Injuries**			**Enough space**		
Gender						
Female	1.34	1.11–1.61	0.002	1.43	1.19–1.73	0.000
Undisclosed	1.05	0.69–1.58	0.819	1.23	0.81–1.86	0.336
Male	Reference category					
Education						
Elementary school or below	0.36	0.23–0.55	0.000	0.32	0.21–0.49	0.000
Junior high school	0.50	0.35–0.71	0.000	0.37	0.26–0.52	0.000
High school	0.73	0.56–0.95	0.018	0.52	0.40–0.67	0.000
Technical school	0.36	0.27–0.49	0.000	0.34	0.25–0.46	0.000
Postgraduate	1.13	0.80–1.61	0.480	1.11	0.78–1.59	0.555
University	Reference category					
Nomadic relations						
Great-grandparents or earlier	0.904	0.68–1.20	0.481	1.118	0.84–1.48	0.442
Grandparents	1.022	0.78–1.34	0.872	0.798	0.61–1.04	0.098
Parents	0.935	0.73–1.20	0.602	0.955	0.74–1.23	0.720
Self or relatives of the same generation	0.624	0.46–0.84	0.002	0.706	0.52–0.95	0.023
No nomadic connections	Reference category					
	**Humane management**			**Natural instincts and behavioral expression**		
Gender						
Female	1.17	0.97–1.42	0.100	1.10	0.91–1.33	0.323
Undisclosed	0.96	0.63–1.45	0.836	0.89	0.59–1.34	0.570
Male	Reference category					
Education						
Elementary school or below	0.46	0.3–0.71	0.000	0.37	0.24–0.56	0.000
Junior high school	0.42	0.29–0.59	0.000	0.40	0.28–0.56	0.000
High school	0.66	0.51–0.86	0.002	0.76	0.58–0.99	0.045
Technical school	0.32	0.24–0.43	0.000	0.38	0.28–0.51	0.000
Postgraduate	1.47	1.02–2.12	0.040	1.10	0.77–1.58	0.585
University	Reference category					
Nomadic relations						
Great-grandparents or earlier	1.091	0.82–1.45	0.550	1.188	0.89–1.58	0.236
Grandparents	1.278	0.97–1.68	0.081	1.274	0.97–1.68	0.084
Parents	0.907	0.70–1.17	0.446	0.968	0.75–1.24	0.797
Self or relatives of the same generation	0.578	0.43–0.78	0.000	0.724	0.54–0.98	0.035
No nomadic connections	Reference category					
	**Comfortable environment**			**No fear**		
Gender						
Female	1.27	1.05–1.54	0.013	1.28	1.06–1.55	0.010
Undisclosed	0.97	0.64–1.46	0.875	0.76	0.50–1.15	0.190
Male	Reference category					
Education						
Elementary school or below	0.34	0.22–0.52	0.000	0.33	0.21–0.51	0.000
Junior high school	0.39	0.28–0.56	0.000	0.41	0.29–0.58	0.000
High school	0.56	0.43–0.73	0.000	0.72	0.56–0.94	0.017
Technical school	0.34	0.25–0.46	0.000	0.29	0.21–0.39	0.000
Postgraduate	0.97	0.68–1.39	0.880	1.21	0.84–1.73	0.307
University	Reference category					
Nomadic relations						
Great-grandparents or earlier	1.147	0.86–1.53	0.348	1.150	0.87–1.53	0.334
Grandparents	1.087	0.83–1.43	0.551	1.489	1.13–1.96	0.005
Parents	0.878	0.68–1.13	0.313	1.113	0.86–1.43	0.408
Self or relatives of the same generation	0.603	0.45–0.81	0.001	0.601	0.45–0.81	0.001
No nomadic connections	Reference category					
	**Clean and safe drinking water**			**No pain**		
Gender						
Female	1.32	1.09–1.60	0.005	1.25	1.03–1.51	0.022
Undisclosed	0.89	0.59–1.35	0.591	0.74	0.49–1.12	0.155
Male	Reference category					
Education						
Elementary school or below	0.26	0.17–0.4	0.000	0.25	0.16–0.38	0.000
Junior high school	0.40	0.28–0.56	0.000	0.41	0.29–0.58	0.000
High school	0.71	0.55–0.93	0.013	0.65	0.50–0.85	0.002
Technical school	0.33	0.24–0.44	0.000	0.33	0.24–0.44	0.000
Postgraduate	1.06	0.74–1.52	0.745	1.14	0.80–1.63	0.464
University	Reference category					
Nomadic relations						
Great-grandparents or earlier	1.121	0.84–1.49	0.436	1.011	0.76–1.34	0.937
Grandparents	1.386	1.05–1.83	0.022	1.527	1.16–2.02	0.003
Parents	0.885	0.69–1.14	0.346	1.017	0.79–1.31	0.897
Self or relatives of the same generation	0.641	0.47–0.86	0.004	0.784	0.58–1.06	0.112
No nomadic connections	Reference category					
	**Feed and Nutrition**					
Gender						
Female	1.16	0.97–1.40	0.109			
Undisclosed	0.95	0.63–1.43	0.792			
Male	Reference category					
Education						
Elementary school or below	0.31	0.20–0.48	0.000			
Junior high school	0.51	0.36–0.72	0.000			
High school	0.57	0.44–0.74	0.000			
Technical school	0.39	0.29–0.53	0.000			
Postgraduate	0.97	0.69–1.37	0.873			
University	Reference category					
Nomadic relations						
Great-grandparents or earlier	1.161	0.88–1.54	0.297			
Grandparents	1.169	0.89–1.53	0.255			
Parents	0.996	0.78–1.28	0.973			
Self or relatives of the same generation	0.722	0.54–0.97	0.032			
No nomadic connections	Reference category					
**Q4: How important are the following conditions in animal care?**						
**Variable**	**OR**	**95% CI**	***p*** **-Value**	**OR**	**95% CI**	***p*** **-Value**
	**Animal farms should have relevant permits for animal care.**			**Animals should be completely dead before being cooked.**		
Gender						
Female	1.42	1.18–1.71	0.000	1.15	0.95–1.39	0.140
Undisclosed	0.94	0.63–1.42	0.787	0.93	0.62–1.40	0.727
Male	Reference category					
Education						
Elementary school or below	0.33	0.22–0.51	0.000	0.37	0.24–0.58	0.000
Junior high school	0.57	0.40–0.81	0.002	0.55	0.39–0.78	0.001
High school	0.65	0.50–0.84	0.001	0.73	0.56–0.94	0.017
Technical school	0.38	0.28–0.51	0.000	0.43	0.32–0.58	0.000
Postgraduate	0.88	0.63–1.25	0.487	0.87	0.62–1.23	0.426
University	Reference category					
Nomadic relations						
Great-grandparents or earlier	1.179	0.89–1.57	0.256	1.105	0.83–1.46	0.486
Grandparents	0.831	0.64–1.09	0.176	1.134	0.87–1.49	0.363
Parents	0.842	0.66–1.08	0.180	0.946	0.74–1.22	0.664
Self or relatives of the same generation	0.618	0.46–0.83	0.002	0.638	0.47–0.86	0.003
No nomadic connections	Reference category					
	**Procedures performed on animals such as ear tagging, castration, and tail docking are acceptable for management.**			**Farm animals should be provided with a comfortable and pleasant living environment.**		
Gender						
Female	0.92	0.76–1.11	0.366	1.11	0.92–1.34	0.290
Undisclosed	0.53	0.35–0.81	0.003	0.78	0.51–1.17	0.224
Male	Reference category					
Education						
Elementary school or below	0.84	0.54–1.30	0.438	0.23	0.15–0.35	0.000
Junior high school	0.74	0.52–1.06	0.100	0.59	0.41–0.83	0.003
High school	0.78	0.60–1.01	0.061	0.74	0.57–0.97	0.028
Technical school	0.82	0.61–1.11	0.207	0.45	0.33–0.61	0.000
Postgraduate	1.18	0.83–1.66	0.357	1.21	0.85–1.73	0.283
University	Reference category					
Nomadic relations						
Great-grandparents or earlier	1.218	0.92–1.61	0.163	1.060	0.80–1.41	0.684
Grandparents	1.612	1.23–2.11	0.001	1.200	0.91–1.58	0.189
Parents	1.051	0.82–1.35	0.693	0.906	0.71–1.16	0.442
Self or relatives of the same generation	1.156	0.86–1.56	0.343	0.623	0.46–0.84	0.002
No nomadic connections	Reference category					
**Q5: Indicate your level of agreement with the following statements.**						
**Variable**	**OR**	**95% CI**	***p*** **-Value**	**OR**	**95% CI**	***p*** **-Value**
	**Animal products should not be purchased if they come from animals that have suffered, even when the products themselves are satisfactory.**			**The transportation time of live animals should be minimized as much as possible.**		
Gender						
Female	1.21	1.00–1.44	0.044	0.97	0.81–1.17	0.783
Undisclosed	1.30	0.87–1.95	0.195	0.93	0.62–1.40	0.730
Male	Reference category					
Education						
Elementary school or below	1.72	1.13–2.63	0.012	0.26	0.17–0.40	0.000
Junior high school	0.94	0.66–1.33	0.720	0.52	0.37–0.74	0.000
High school	1.25	0.97–1.61	0.088	0.52	0.40–0.67	0.000
Technical school	1.09	0.81–1.46	0.577	0.34	0.25–0.45	0.000
Postgraduate	1.02	0.73–1.42	0.909	1.06	0.75–1.50	0.758
University	Reference category					
Nomadic relations						
Great-grandparents or earlier	0.757	0.58–0.99	0.045	1.049	0.79–1.39	0.737
Grandparents	0.863	0.67–1.12	0.268	0.962	0.74–1.26	0.776
Parents	0.829	0.65–1.06	0.130	0.988	0.77–1.27	0.925
Self or relatives of the same generation	1.006	0.75–1.35	0.966	0.713	0.53–0.96	0.026
No nomadic connections	Reference category					
	**Animal products should not be purchased if they come from animals that have suffered, even when the products have low price**			**Legislation to ensure animal protection is important.**		
Gender						
Female	1.20	1.00–1.44	0.045	1.41	1.16–1.70	0.000
Undisclosed	1.67	1.12–2.49	0.012	0.93	0.62–1.41	0.746
Male	Reference category					
Education						
Elementary school or below	1.87	1.22–2.85	0.004	0.22	0.14–0.33	0.000
Junior high school	1.01	0.71–1.42	0.961	0.41	0.29–0.58	0.000
High school	0.90	0.70–1.17	0.442	0.50	0.38–0.65	0.000
Technical school	1.02	0.75–1.37	0.919	0.35	0.26–0.48	0.000
Postgraduate	1.22	0.88–1.7	0.232	1.09	0.76–1.57	0.621
University	Reference category					
Nomadic relations						
Great-grandparents or earlier	0.646	0.49–0.85	0.002	1.005	0.76–1.33	0.971
Grandparents	0.682	0.53–0.89	0.004	1.071	0.82–1.41	0.624
Parents	0.835	0.66–1.06	0.143	0.957	0.74–1.23	0.733
Self or relatives of the same generation	1.034	0.77–1.38	0.819	0.763	0.56–1.03	0.078
No nomadic connections	Reference category					
	**Animals should be unconscious before being slaughtered.**			**Animal protection organizations are very important in ensuring that animals are well cared for.**		
Gender						
Female	0.80	0.67–0.96	0.017	1.28	1.06–1.54	0.010
Undisclosed	0.78	0.52–1.17	0.232	0.83	0.55–1.25	0.374
Male	Reference category					
Education						
Elementary school or below	0.47	0.31–0.72	0.001	0.43	0.28–0.67	0.000
Junior high school	0.70	0.49–0.98	0.039	0.52	0.36–0.73	0.000
High school	0.99	0.76–1.28	0.914	0.75	0.57–0.97	0.028
Technical school	0.61	0.45–0.82	0.001	0.36	0.27–0.49	0.000
Postgraduate	1.34	0.95–1.89	0.097	0.89	0.63–1.26	0.517
University	Reference category					
Nomadic relations						
Great-grandparents or earlier	0.952	0.72–1.25	0.723	0.999	0.75–1.32	0.993
Grandparents	1.302	1.00–1.7	0.054	0.813	0.62–1.06	0.129
Parents	0.828	0.65–1.06	0.132	0.902	0.70–1.16	0.423
Self or relatives of the same generation	0.655	0.49–0.88	0.005	0.628	0.47–0.85	0.002
No nomadic connections	Reference category					

### 3.4. Motivation for Caring for Animals

We further examined respondents’ motivations for caring about farm animal welfare (Table 7), including utilitarian, emotional, spiritual, religious, equality-based, and socio-cultural reasons. Overall, gender and education level again emerged as the most consistent predictors, whereas the effects of nomadic background varied by generational proximity.

Females were more likely think that it is important to treat animals well because of personal love and compassion for animals, because animals have spirituality, because animals are equal to humans, and because of their customs or family education, but not because of their contribution to humanity, their religious belief, or for better livestock products.

Compared with university graduates, elementary, junior high, and technical school leavers were less likely to think it important to treat animals well for all posited reasons, except for their own religious beliefs, which only motivated respondents with elementary and technical school leavers to care less about treating animals well. High school leavers were only motivated to care less because of their beliefs about animals’ contribution to humanity, their personal love and compassion for animals, and because animals have spirituality. Postgraduates did not differ from graduates in their beliefs. Thus, the principal educational divide lies between tertiary and non-tertiary education rather than within higher education levels.

The main influence of nomadic connections on the importance of treating animals well was on respondents’ beliefs about animals’ spirituality and their own religious beliefs. Compared with respondents with no nomadic connections, respondents who themselves or their relatives of the same generation were nomadic were less likely to believe that better livestock products or animals’ spirituality are reasons to treat farm animals well. However, respondents whose ancestors of any generation were nomadic were more likely to believe that animals’ spirituality is a reason to treat animals well, even though this did not extend to the current generation. Similarly, respondents whose grandparents were nomadic were more likely to believe that animals’ spirituality is a reason to treat animals well.

In summary, the regression analysis results demonstrate the influences of gender, education, and nomadic connections on attitudes and perceptions regarding animal welfare. Women and highly educated groups were more supportive of modern animal welfare concepts, while the influence of nomadic connections showed significant generational differences.

## 4. Discussion

According to Cochran’s formula, at a 95% confidence level, even with an infinitely large population, a sample size of around 1000–1100 can control the error within ±3%. In large-scale national surveys, a sample size of 1000–2000 represents the optimal range for balancing statistical power and cost. Therefore, the data from the 1660 questionnaires presented in this paper are sufficiently statistically representative [20].

Based on 1660 valid responses, this study reveals a multidimensional picture of Chinese public attitudes towards animal welfare, analyzed through gender, education level, and nomadic connections. Overall, the findings suggest that the concept of animal welfare is becoming better understood in Chinese society, with nearly two-thirds of respondents saying they were familiar with the concept and the vast majority of respondents agreeing with the need to treat animals kindly. This finding echoes the emphasis placed on the construction of an ecologically focused civilization in Chinese society in recent years [21].

Public support for animal welfare was strong, and 73% of respondents said they were willing to pay a premium, but this willingness to pay was mainly concentrated in the low range (5–10%). Over the past few decades, the primary objective of China’s agriculture sector has been to ensure an adequate food supply. In recent years, policy priorities have gradually shifted towards improving quality, efficiency, and green development. And since 2017, the construction of an ecologically focused civilization has been established as the fundamental principle of development, but animal welfare is still regarded as a preferred rather than a core attribute [21,22]. Consumers who support animal welfare products equate high welfare with safer, healthier products with high quality. The public expects better products, but due to a series of imperfect measures such as the imperfect market certification system, when faced with economic trade-offs, consumers are still limited by price in terms of actual purchasing power, although they recognize high-welfare products ideologically [23,24]. This phenomenon of high verbal support and low actual payment, in addition to economic constraints, may also stem from the potential that respondents are affected by social expectation bias in questionnaires, which refers to the bias of respondents to answer questions based on others’ approval rather than true attitudes or behaviors [25].

Gender

Gender is the most stable variable in predicting attitudes towards animal welfare. Consistent with a large number of international studies, this research found that women have significantly more positive attitudes than men across almost all dimensions [26,27,28]. This is not only reflected in their focus on basic survival needs such as clean drinking water and adequate food, but also in their emphasis on the mental state of animals (such as animal management without pain or fear). But behind this phenomenon lie different ethical driving forces in Chinese and Western contexts. In the West, women’s attitudes towards animal welfare are often influenced by the public’s political awareness of animal rights, manifesting in their support for the independent rights of animals and emphasizing animals as individuals with intrinsic value and independent moral standing [29,30]. This support primarily stems from the pursuit of social justice, emphasizing that animals are individuals with independent rights. Women’s focus on animal welfare is also closely related to their values regarding social equality and human rights. Women’s comprehensive leadership in animal welfare attitudes is closely related to the current trend of emotional socialization in China. In modern Chinese families, women are often the primary decision-makers in purchasing, a concept known as “her economy,” which was proposed by economist Shi Qingqi in 2007 [31]. It refers to the unique economic circle and phenomenon formed by female consumption [32]. According to a survey by consulting firm Accenture, China has nearly 400 million female consumers aged between 20 and 60, who control an annual consumption expenditure of as high as 1 trillion Chinese Yuan, dominating, for example, the pet ownership sector. In previous studies, pet ownership has been found to influence participants’ attitudes towards animal welfare and further enhance related awareness [33,34]. With rapid urbanization, pets in China have increasingly shifted from functional roles, such as guarding property, to becoming important sources of emotional companionship. This interspecies intimacy may facilitate the extension of empathy beyond pets to other categories of animals [28].

This difference may also be related to social role theory. Women in traditional societies are often socialized to have stronger nurturing traits and greater affinity, making them more prone to empathize with vulnerable groups, including animals [35,36]. This is probably the reason why women had more positive attitudes towards female animals kept for breeding, such as cows, sheep, and laying hens, but not beef cattle or broiler chickens that are raised to be slaughtered for meat. In contrast, men in traditional societies tend to develop a more instrumental perspective. This does not mean men lack care, but rather they are more accustomed to viewing animals as production materials rather than emotional projections. This may be related to the theory of hegemonic masculinity, a traditional psychological model of masculinity based on power and dominance, which suggests that men should appear rough and not express emotions [37]. In this context, excessive display of sympathy for animals is often seen as weakness or unmasculine/feminine; so, men tend to view animals as production materials or resources to meet societal expectations of male roles [38]. Ongoing shifts in gender norms may gradually reshape how both men and women articulate their views on animal welfare.

Education level

Higher levels of education were generally associated with stronger awareness and support for animal welfare, benefiting from increasingly accessible information about animal welfare and the popularization of general education in universities. Researchers generally believe that the higher the level of education, the greater the exposure to modern civilized concepts and the stronger the support for animal welfare. In most survey items of this study, respondents with a college education or above exhibited more positive attitudes and a deeper understanding of animal welfare. Education can shape cognition, build a moral framework, and enhance empathy, making those with higher education more likely to engage with ethics, ecological science, and updated animal welfare issues, thereby forming a more stable interspecies care consciousness, tending to view animal welfare as an expression of personal moral cultivation and civilization, and further extending moral concern to species beyond humans [2,39,40,41,42,43]. However, this positive association was not universal. Our data show that respondents with an elementary school education or below were more likely than those with a university degree to strongly oppose management practices such as ear tagging and castration, which involve direct pain. This finding suggests that educational differences may reflect distinct evaluative approaches rather than simple differences in moral concern.

Highly educated individuals, especially those exposed to knowledge related to animal welfare, may have cognitively accepted the logic of modern intensive farming. They understand that while practices like castration and ear tagging cause short-term pain, they are necessary for managing livestock in economic animal farming, improving meat quality, and avoiding conflicts during mating seasons [44,45]. Alternatively, they may be skeptical about the feasibility of fully tracing and boycotting such practices within vast market networks [44,46]. In contrast, groups with lower educational attainment found it difficult to establish the legitimacy of animal management methods and may rely more on intuitive morality. Without framing these practices within industrial or economic rationales, they may be more inclined to reject them on the basis of immediate suffering. Being influenced by traditional beliefs such as animism or karmic retribution, which respect the integrity of an animal’s body and view artificial management practices like tail docking as unnatural and even cruel [8], they may hold the view that one should not purchase animals if they are suffering.

Nomadic connections

The data from this study show that for many parameters that we studied, the relationship between nomadic connections and animal welfare attitudes is complex and non-linear. Individuals who themselves—or whose same-generation relatives—have a nomadic background generally reported lower levels of welfare awareness across several domains. In contrast, respondents with more distant nomadic ancestry at the grandparent level especially demonstrated heightened concern in selected areas, particularly regarding animal pain, fear, water quality, and religious or spiritual justifications for animal care.

The group without nomadic connections scored higher than the nomadic group on multiple animal welfare attitude measures. This finding is consistent with mainstream international research on urban–rural differences, which indicates that urban residents farther from agricultural production often exhibit more positive attitudes towards animal welfare [47,48,49]. This contradicts the stereotype of nomadic people’s love for nature and reflects changes in the relationship between humans and animals in modern society. For this reason, we suspect that there has been a generational change in the attitudes of people with nomadic connections towards animals. For participants without any nomadic connection, animals may primarily exist as pets or abstract objects of conservation, and their perception of animals may be less shaped by production realities [50].

For individuals with a nomadic connection, particularly those who themselves have the connection or family members of the same generation, animals are likely to be closely tied to their livelihood [51]. As the present data show, they answered more pragmatically and negatively on items involving actual production operations. Their scores on related ethical indicators were relatively low. This should be understood as a livelihood-driven adaptation. With the implementation of grazing return-to-grass policies, the promotion of large-scale farming, and the market’s extreme pursuit of slaughter rates, modern herders, as operators, need to address feed costs, disease risks, and market fluctuations and therefore have a very different relationship to animals compared with their ancestors [52,53]. In addition to these ongoing structural changes, modern herders have to manage and face more challenges such as the expansion of large-scale farming and market pressures to maximize productivity. Livestock industry practitioners need to use psychological defense mechanisms to cope with work in daily feeding, transportation, and slaughter. If they invest too much emotion in every animal, they will not be able to bear the psychological burden of killing animals. Therefore, they must psychologically objectify animals to reduce moral anxiety [54]. This is a common psychological adaptation mechanism among livestock industry practitioners, not necessarily a lack of morality [55].

Participants with nomadic ancestry in their grandparents’ generation had some significantly better attitudes towards animal welfare than those currently engaged in nomadic lifestyles and even outperformed the control group on certain issues. Although these respondents are no longer engaged in nomadism, the traditional nomadic cultural characteristics might have evolved into an underlying cultural logic that influences the relationship between humans and livestock. Traditional Mongolian and other nomadic cultures contain simple ecological ethics. In that era, two generations prior, livestock were considered family members, and humans and animals existed in a symbiotic relationship, with traditions such as not killing young animals, not causing them to be startled, and the belief that all things have spirits. This generation has learned reverence for animals through storytelling from their elders or festive rituals, but they did not inherit the survival pressures/skills of their grandparents in harsh natural environments. Therefore, they can preserve the compassionate aspects of the nomadic culture while filtering out the harshness of herding. In the waves of modernization and urbanization, individuals with the heritage of nomadic culture living in cities tend to psychologically maintain the idealized image of traditional nomadic culture’s kindness to animals to reinforce their ethnic or cultural identity. Traditional nomadic ecology has the potential to be combined with ecological civilization construction, promoting indigenous animal welfare education based on local cultural identity rather than solely from a Western perspective [56].

Limitations

There are several limitations that warrant consideration. First, due to the use of online questionnaires, the sample shows the characteristics of youth and relatively high education, which may not fully represent the true attitudes of people in China’s remote rural areas, especially the elderly herders. Second, it is difficult to completely avoid the bias of social expectations in questionnaires and respondents may, for example, overestimate their willingness to pay. Future research could adopt experimental approaches, such as real purchase simulations, to observe actual payment behavior under conditions involving real monetary transactions. Alternatively, future research should incorporate behavioral indicators—for example, asking participants how often they actually purchase high-priced welfare products. Third, self-reported nomadic heritage may be subject to bidirectional bias, under-reporting due to urbanization and social stigma, or over-reporting driven by cultural identity and ancestral pride. Furthermore, regarding key variables, nomadic connections, this study categorized respondents based on kinship and generational distance. While this provided a quantifiable metric for intergenerational cultural transmission, we acknowledge that it may not explicitly measure the intensity or quality of personal interactions with the relatives. Future research should refine this variable, for example, by incorporating Likert-scale questions and asking respondents to what extent the relatives shaped their attitudes towards animals.

In addition, cross-sectional design limits the ability to draw causal inferences. This study cannot determine how attitudes towards animal welfare evolve as individuals shift away from nomadic livelihoods or across generations. Longitudinal or mixed-method studies would be valuable in clarifying how changing socioeconomic contexts shape the development of animal welfare perceptions over time. Finally, while this study provides comprehensive insights into public attitudes towards animal welfare, it did not empirically measure actual husbandry practices or welfare-related behaviors. Future research can further explore how these inconsistencies may hinder the implementation of welfare standards through simulation experiments, and how factors influence this process.

## 5. Conclusions

We found that the attitudes of the Chinese public towards animal welfare showed a positive trend, which is probably driven by factors such as higher education levels and gender, both of which were highly influential in our survey. Women and those with higher education levels exhibited more positive attitudes towards most aspects of animal welfare, and women were more benign towards farm animals that are kept for reproduction, but not animals kept for meat. Respondents with lower education levels showed stronger resistance to invasive rearing management methods. The influence of nomadic connections on animal welfare was complex; participants with personal or same-generation nomadic connections tended to have more negative attitudes towards animals. In contrast, groups with more distant nomadic connections in their ancestors had retained traditional ecological ethical concepts in selected domains., and this attitude may be modulated by economic rationality and livelihood pressures. Understanding these nuanced differences will be of great significance for animal welfare education and promotion in Chinese people.

## Figures and Tables

**Table 1:** Independent assessment of three key variables: gender, education, and nomadic lifestyle (N = 1660).

Spearman’s		Gender	Nomadic Lifestyle	Education
Gender	Rho	1	−0.061 *	0.030
	*p*		0.013	0.222
Nomadic Lifestyle	Rho	−0.061 *	1	0.033
	*p*	0.013		0.181
	N	1660	1660	1660
Education	Rho	0.030	0.033	1
	*p*	0.222	0.181	

Note: * *p* < 0.05.

**Table 2:** Independent assessment of two variables: region and nomadic lifestyle (N = 1660).

Spearman’s		Nomadic Lifestyle	Region
Nomadic Lifestyle	Rho	1	0.089 **
	*p*		0.001
Region	Rho	0.089 **	1
	*p*	0.001	

Note: ** *p* < 0.01.

**Table 3:** Socio-demographic data of the respondents.

Basic Information on Socio-Demographics			
Variable	Option	N	%
Gender	Male	856	51.6
	Female	718	43.3
	Not Undisclosed	86	5.2
Age	18–24	766	46.1
	25–34	362	21.8
	35–44	230	13.9
	45–54	158	9.5
	55–64	93	5.6
	>65	51	3.1
Ethnic group	Han	902	54.3
	Mongolian	591	35.6
	Hui	49	3.0
	Uyghur	23	1.4
	Tibetan	24	1.5
	Manchu	35	2.1
	Oroqen Tribe	8	0.5
	Daur	5	0.3
	Evenki	4	0.2
	Korean	7	0.4
	Other	12	0.7
Educational level	University graduate	911	54.9
	High school	251	15.1
	Technical school	170	10.2
	Postgraduate	131	7.9
	Junior high school	122	7.4
	Elementary school or below	75	4.5
Occupation	Livestock husbandry	232	14.0
	Agriculture	197	11.9
	Education	175	10.5
	Arts	112	6.8
	Health	95	5.7
	Technology	91	5.5
	Finance	89	5.4
	Science	81	4.9
	Administration	69	4.2
	Construction	64	3.9
	Sales	64	3.9
	Logistics	57	3.4
	Mining	52	3.1
	Other	282	17.0
Place of residence	Urban	670	40.4
	Rural	494	30.0
	Village	438	26.4
	Other	58	3.4
Nomadic connections	None	524	31.6
	Self or family members of same generation	210	12.7
	Parents	367	22.1
	Grandparents	300	18.1
	Great-grandparents or earlier	259	15.6

**Table 4:** Responses to questions related to animal welfare issues.

Respondents’ Basic Understanding of Animal Welfare			
Question	Option	N	%
How familiar are you with the term animal welfare?	Very familiar	532	32.1
	Familiar	527	31.8
	Neither familiar nor unfamiliar	377	22.7
	Not very familiar	145	8.7
	Not familiar	79	4.8
Do you live in harmony with animals?	Very harmonious	727	43.8
	Acceptable	507	30.5
	Average	258	15.5
	Not very acceptable	93	5.6
	Disgust	75	4.5
Do you think it is important to treat animals kindly?	Very important	870	52.4
	Important	389	23.4
	Neither important nor not important	221	13.3
	Not very important	106	6.4
	Not important	74	4.5
How necessary do you think it is to promote humane treatment of livestock and poultry?	Very necessary	817	49.2
	Necessary	438	26.4
	Neither necessary nor not necessary	235	14.2
	Not very necessary	111	6.7
	Not necessary	59	3.6
Are you willing to pay a higher price for products from animals that have received better care?	Yes	1208	72.8
	No	452	27.2
How much extra are you willing to pay if you choose to?	5%	296	24.5
	10%	276	22.9
	20%	322	26.7
	50%	150	12.4
	100%	79	6.5
	>100%	85	7.0

**Table 5:** Significant effects of gender, education and nomadic relations on respondents’ attitudes towards animals.

Part 1						
**Q1: To What Extent Do You Think Different Types of Animals Should Be Treated Kindly?**						
**Variable**	**OR**	**95% CI**	***p*** **-Value**	**OR**	**95% CI**	***p*** **-Value**
	**Pets**			**Abandoned Pets**		
Gender						
Female	1.42	1.17–1.71	0.000	1.46	1.21–1.77	0.000
Undisclosed	1.12	0.74–1.69	0.593	0.93	0.62–1.41	0.744
Male	Reference category					
Education						
Elementary school or below	0.35	0.22–0.53	0.000	0.41	0.26–0.63	0.000
Junior high school	0.43	0.30–0.60	0.000	0.37	0.26–0.52	0.000
High school	0.79	0.60–1.02	0.073	0.84	0.64–1.09	0.185
Technical school	0.35	0.26–0.47	0.000	0.37	0.27–0.50	0.000
Postgraduate	1.00	0.70–1.41	0.986	0.81	0.58–1.15	0.241
University	Reference category					
Nomadic relations						
Great-grandparents or earlier	0.92	0.70–1.22	0.568	1.02	0.77–1.35	0.893
Grandparents	0.91	0.70–1.20	0.509	0.85	0.65–1.11	0.236
Parents	0.72	0.56–0.93	0.011	1.05	0.81–1.35	0.729
Self or relatives of the same generation	0.61	0.46–0.83	0.001	0.58	0.43–0.78	0.000
No nomadic connections	Reference category					
	**Laboratory animals**			**Wildlife**		
Gender						
Female	1.29	1.07–1.56	0.007	1.35	1.12–1.63	0.002
Undisclosed	1.08	0.72–1.63	0.703	0.97	0.64–1.46	0.870
Male	Reference category					
Education						
Elementary school or below	0.40	0.26–0.61	0.000	0.37	0.24–0.57	0.000
Junior high school	0.38	0.27–0.53	0.000	0.43	0.3–0.61	0.000
High school	0.62	0.48–0.8	0.000	0.55	0.42–0.71	0.000
Technical school	0.50	0.37–0.68	0.000	0.31	0.23–0.42	0.000
Postgraduate	1.02	0.72–1.44	0.903	0.73	0.52–1.02	0.068
University	Reference category					
Nomadic relations						
Great-grandparents or earlier	1.02	0.77–1.35	0.882	1.12	0.84–1.48	0.448
Grandparents	1.22	0.93–1.59	0.152	1.13	0.86–1.48	0.384
Parents	0.89	0.69–1.14	0.353	1.01	0.79–1.3	0.910
Self or relatives of the same generation	0.61	0.46–0.82	0.001	0.79	0.58–1.06	0.117
No nomadic connections	Reference category					
	**Farm animals**					
Gender						
Female	1.47	1.22–1.78	0.000			
Undisclosed	0.98	0.65–1.47	0.906			
Male	Reference category					
Education						
Elementary school or below	0.25	0.16–0.38	0.000			
Junior high school	0.53	0.37–0.75	0.000			
High school	0.69	0.53–0.9	0.006			
Technical school	0.32	0.23–0.43	0.000			
Postgraduate	0.72	0.52–1.02	0.065			
University	Reference category					
Nomadic relations						
Great-grandparents or earlier	0.98	0.74–1.29	0.884			
Grandparents	1.34	1.02–1.76	0.034			
Parents	1.10	0.86–1.41	0.458			
Self or relatives of the same generation	0.86	0.64–1.16	0.327			
No nomadic connections	Reference category					
**Q2: To what extent do you think different types of farm animals should be treated kindly?**						
**Variable**	**OR**	**95% CI**	***p*** **-Value**	**OR**	**95% CI**	***p*** **-Value**
	**Cow**			**Broiler chicken**		
Gender						
Female	1.27	1.05–1.53	0.013	1.15	0.95–1.39	0.146
Undisclosed	1.14	0.75–1.73	0.540	0.87	0.58–1.31	0.502
Male	Reference category					
Education						
Elementary school or below	0.34	0.22–0.52	0.000	0.34	0.22–0.53	0.000
Junior high school	0.42	0.30–0.60	0.000	0.39	0.27–0.55	0.000
High school	0.65	0.50–0.85	0.001	0.74	0.57–0.97	0.027
Technical school	0.33	0.25–0.45	0.000	0.47	0.34–0.63	0.000
Postgraduate	1.45	1.00–2.09	0.048	0.99	0.70–1.41	0.972
University	Reference category					
Nomadic relations						
Great-grandparents or earlier	1.16	0.87–1.54	0.315	1.03	0.78–1.37	0.836
Grandparents	0.93	0.71–1.21	0.580	1.19	0.91–1.56	0.211
Parents	1.03	0.80–1.33	0.804	1.00	0.78–1.29	0.978
Self or relatives of the same generation	0.84	0.62–1.14	0.270	0.65	0.48–0.88	0.005
No nomadic connections	Reference category					
	**Beef cattle**			**Pig**		
Gender						
Female	1.21	1.00–1.46	0.051	1.43	1.18–1.72	0.000
Undisclosed	1.09	0.72–1.65	0.692	1.08	0.72–1.63	0.707
Male	Reference category					
Education						
Elementary school or below	0.25	0.16–0.39	0.000	0.34	0.22–0.53	0.000
Junior high school	0.48	0.34–0.69	0.000	0.60	0.42–0.85	0.004
High school	0.68	0.52–0.88	0.004	0.70	0.54–0.91	0.008
Technical school	0.25	0.19–0.34	0.000	0.43	0.32–0.58	0.000
Postgraduate	0.84	0.60–1.19	0.332	1.01	0.71–1.42	0.967
University	Reference category					
Nomadic relations						
Great-grandparents or earlier	1.06	0.80–1.40	0.703	1.06	0.80–1.41	0.664
Grandparents	1.18	0.90–1.55	0.237	0.89	0.68–1.16	0.397
Parents	1.01	0.78–1.29	0.963	0.96	0.75–1.24	0.767
Self or relatives of the same generation	0.75	0.56–1.02	0.065	0.70	0.52–0.94	0.018
No nomadic connections	Reference category					
	**Sheep**			**Camel**		
Gender						
Female	1.29	1.07–1.56	0.009	1.16	0.97–1.41	0.111
Undisclosed	0.89	0.59–1.35	0.594	0.96	0.63–1.45	0.840
Male	Reference category					
Education						
Elementary school or below	0.48	0.31–0.74	0.001	0.29	0.19–0.45	0.000
Junior high school	0.53	0.38–0.76	0.000	0.34	0.24–0.48	0.000
High school	0.84	0.65–1.10	0.210	0.65	0.50–0.85	0.001
Technical school	0.43	0.32–0.58	0.000	0.34	0.25–0.45	0.000
Postgraduate	1.09	0.77–1.55	0.638	0.84	0.60–1.19	0.329
University	Reference category					
Nomadic relations						
Great-grandparents or earlier	0.93	0.70–1.22	0.586	1.07	0.81–1.42	0.620
Grandparents	1.47	1.11–1.94	0.006	1.22	0.93–1.59	0.157
Parents	1.03	0.80–1.32	0.835	1.10	0.86–1.42	0.451
Self or relatives of the same generation	0.55	0.41–0.74	0.000	0.74	0.55–0.99	0.043
No nomadic connections	Reference category					
	**Goat**			**Horse**		
Gender						
Female	1.42	1.18–1.72	0.000	1.19	0.99–1.44	0.071
Undisclosed	0.97	0.64–1.46	0.871	0.84	0.55–1.26	0.394
Male	Reference category					
Education						
Elementary school or below	0.34	0.22–0.53	0.000	0.31	0.20–0.48	0.000
Junior high school	0.58	0.41–0.83	0.003	0.55	0.39–0.79	0.001
High school	0.72	0.55–0.93	0.014	0.83	0.64–1.09	0.180
Technical school	0.37	0.28–0.5	0.000	0.33	0.24–0.44	0.000
Postgraduate	1.03	0.73–1.46	0.870	0.80	0.56–1.13	0.198
University	Reference category					
Nomadic relations						
Great-grandparents or earlier	1.05	0.79–1.39	0.736	0.89	0.67–1.18	0.432
Grandparents	0.80	0.61–1.05	0.102	1.17	0.89–1.54	0.263
Parents	0.98	0.76–1.26	0.860	0.96	0.74–1.24	0.757
Self or relatives of the same generation	0.64	0.47–0.86	0.003	0.63	0.46–0.84	0.002
No nomadic connections	Reference category					
	**Laying hen**			**Donkey**		
Gender						
Female	1.41	1.17–1.70	0.000	1.21	1.00–1.46	0.050
Undisclosed	1.35	0.89–2.05	0.157	0.92	0.61–1.40	0.710
Male	Reference category					
Education						
Elementary school or below	0.28	0.18–0.42	0.000	0.41	0.27–0.64	0.000
Junior high school	0.50	0.35–0.71	0.000	0.45	0.32–0.64	0.000
High school	0.57	0.44–0.74	0.000	0.78	0.60–1.01	0.064
Technical school	0.37	0.27–0.50	0.000	0.37	0.27–0.5	0.000
Postgraduate	0.87	0.62–1.23	0.427	0.87	0.62–1.23	0.440
University	Reference category					
Nomadic relations						
Great-grandparents or earlier	0.93	0.70–1.23	0.594	0.97	0.73–1.28	0.840
Grandparents	0.78	0.60–1.02	0.068	1.38	1.05–1.81	0.022
Parents	0.93	0.72–1.19	0.562	1.01	0.79–1.3	0.943
Self or relatives of the same generation	0.57	0.42–0.77	0.000	0.68	0.51–0.92	0.012
No nomadic connections	Reference category					

**Table 7:** Significant effects of gender, education and nomadic relations on respondents’ attitudes towards motivation for caring for animals.

Part 3						
**Q6: Why Do You Think It Is Important to Treat Farm Animals Well?**						
**Variable**	**OR**	**95% CI**	***p*-Value**	**OR**	**95% CI**	***p*-Value**
	**Contribution to Humanity**			**Personal Love and Compassion for Animals**		
Gender						
Female	1.09	0.91–1.32	0.349	1.28	1.06–1.55	0.009
Undisclosed	0.92	0.61–1.39	0.707	0.90	0.6–1.35	0.608
Male	Reference category					
Education						
Elementary school or below	0.35	0.22–0.53	0.000	0.23	0.15–0.35	0.000
Junior high school	0.66	0.47–0.94	0.022	0.42	0.30–0.60	0.000
High school	0.65	0.50–0.85	0.001	0.64	0.49–0.83	0.001
Technical school	0.46	0.34–0.62	0.000	0.28	0.21–0.38	0.000
Postgraduate	0.91	0.65–1.28	0.588	0.75	0.53–1.06	0.103
University	Reference category					
Nomadic relations						
Great-grandparents or earlier	1.22	0.92–1.61	0.164	1.12	0.85–1.48	0.431
Grandparents	0.94	0.72–1.23	0.650	1.19	0.91–1.57	0.199
Parents	0.89	0.69–1.14	0.349	1.00	0.78–1.29	0.989
Self or relatives of the same generation	0.79	0.59–1.07	0.130	0.88	0.65–1.19	0.407
No nomadic connections	Reference category					
	**Animals have spirituality**			**Religious beliefs**		
Gender						
Female	1.26	1.04–1.52	0.016	1.04	0.86–1.24	0.694
Undisclosed	0.94	0.62–1.43	0.785	0.84	0.56–1.26	0.406
Male	Reference category					
Education						
Elementary school or below	0.21	0.14–0.33	0.000	0.60	0.39–0.92	0.018
Junior high school	0.36	0.25–0.51	0.000	0.99	0.70–1.41	0.975
High school	0.62	0.48–0.81	0.000	1.01	0.78–1.31	0.940
Technical school	0.34	0.25–0.46	0.000	0.60	0.45–0.81	0.001
Postgraduate	1.05	0.74–1.5	0.776	0.82	0.59–1.14	0.238
University	Reference category					
Nomadic relations						
Great-grandparents or earlier	0.93	0.71–1.24	0.639	1.46	1.11–1.91	0.006
Grandparents	1.43	1.08–1.89	0.011	2.43	1.86–3.17	0.000
Parents	0.93	0.72–1.19	0.554	1.53	1.20–1.95	0.001
Self or relatives of the same generation	0.57	0.42–0.76	0.000	1.25	0.94–1.68	0.130
No nomadic connections	Reference category					
	**Animals are equal to humans**			**Customs or family education**		
Gender						
Female	1.59	1.32–1.92	0.000	1.33	1.10–1.60	0.003
Undisclosed	0.90	0.60–1.35	0.600	1.09	0.73–1.65	0.672
Male	Reference category					
Education						
Elementary school or below	0.32	0.21–0.50	0.000	0.44	0.28–0.67	0.000
Junior high school	0.58	0.41–0.82	0.002	0.49	0.35–0.70	0.000
High school	0.99	0.76–1.29	0.916	0.95	0.73–1.23	0.694
Technical school	0.42	0.31–0.57	0.000	0.45	0.33–0.60	0.000
Postgraduate	1.26	0.89–1.79	0.199	0.81	0.58–1.14	0.233
University	Reference category					
Nomadic relations						
Great-grandparents or earlier	1.30	0.98–1.72	0.068	1.30	0.99–1.72	0.063
Grandparents	1.29	0.99–1.69	0.064	1.28	0.98–1.68	0.065
Parents	1.09	0.85–1.40	0.513	1.14	0.89–1.47	0.283
Self or relatives of the same generation	0.81	0.60–1.09	0.165	0.97	0.72–1.31	0.856
No nomadic connections	Reference category					
	**For better livestock products**					
Gender						
Female	1.07	0.89–1.29	0.458			
Undisclosed	0.92	0.61–1.39	0.697			
Male	Reference category					
Education						
Elementary school or below	0.65	0.42–1.00	0.049			
Junior high school	0.60	0.42–0.84	0.003			
High school	0.87	0.67–1.13	0.291			
Technical school	0.62	0.46–0.83	0.002			
Postgraduate	1.36	0.96–1.93	0.079			
University	Reference category					
Nomadic relations						
Great-grandparents or earlier	1.34	1.01–1.77	0.040			
Grandparents	1.08	0.83–1.41	0.576			
Parents	0.91	0.71–1.16	0.448			
Self or relatives of the same generation	0.74	0.55–0.99	0.043			
No nomadic connections	Reference category					

## Data Availability

The original contributions presented in this study are included in the article. Further inquiries can be directed to the corresponding authors.

## Appendix A

**1. Do you identify as Chinese?** Yes (please continue); No (if no, please do not continue. Thank you for your time) **2. What is your gender?** Female; Male; Undisclosed. **3. What is your age?** 18–24; 25–34; 35–44; 45–54; 55–64; ≥65. **4. Please indicate whether anyone in your family lives or lived a nomadic life**. no-one, great-grandparents or earlier, grandparents, parents, self, or same-generation relatives **5. What is your ethnic group?** Han; Mongolian; Hui; Uyghur; Tibetan; Manchu; Oroqen Tribe; Daur; Evenki; Korean; Other **6. What is your highest education level?** Elementary school or below; Junior high school; High school; Technical School; University; Postgraduate. **7. What is your occupation?** Livestock-husbandry; Agriculture; Arts; Construction; Finance; Education; Administration; Health; Mining; Technology; Sales; Science; Logistic; Other; **8. What is your region type?** Rural; Village; Urban; Other. **9. How familiar are you with the term animal welfare?** Very familiar; Familiar; Neither familiar nor unfamiliar; Not very familiar; Not familiar. **10. Do you live in harmony with animals?** Very harmonious; Acceptable harmony; Average; Not very acceptable harmony; Disgust at contact with animals. **11. Do you have direct contact with animals? Daily contact; Frequent contact; Occasional contact; Basically no contact; Never any contact**. **12. Why do you have contact with animals because?** Raising Pets; Raising livestock at home; Farm work; Zoo. **13. Do you think it’s important to treat animals kindly?** Very important; Important; Neither important nor unimportant; Not very important; Not important. **14. Where did you receive the awareness to be kind to animals?** Formal Study; Family; Friends; Pastoralists; Government; it formed naturally; Animal protection organizations; Social media. **15. Do you think it is necessary to promote kindness to livestock and poultry?** Very necessary; Necessary; Neither necessary nor unnecessary; Not really necessary; Not necessary at all. **16. Are you willing to pay a higher price for products from animals that have received better care?** Yes; No. **17. How much extra are you willing to pay if you choose to?** 5%; 10%; 20%; 50%; 100%; >100% **18. What do you think is the current standard of animal care?** Very good; Fairly good; Fair; Poor; Very poor. **19. Who do you think is most responsible for the adequate care of animals?** Government; Animal protection organizations; Farmers; All of society; People who like animals; People who own animals; Companies that use animals; Other. **20. Which animal group do you think has a higher level of animal welfare?** **Differentiated into:** **20. 1.** Pets **20. 2.** Laboratory animals **20. 3.** Farm animals **20. 4.** Abandoned pets **20. 5.** Wild animals **21. I think animals should be treated kindly (select the extent to which you agree)** (Strongly disagree; Disagree; Neither agree nor disagree; Agree; Strongly agree) **22. To what extent do you think the following farm animals should be treated kindly?** (Strongly disagree; Disagree; Neither agree nor disagree; Agree; Strongly agree) **22. 1.** Dairy cow **22. 2.** Beef cattle **22. 3.** Sheep **22. 4.** Goat **22. 5.** Laying Chicken **22. 6.** Broiler **22. 7.** Pig **22. 8.** Camel **22. 9.** Horse **22. 10.** Donkey **22. To what extent do you think the following factors affect the welfare of farm animals?** (Very much; to some extent; neither a lot nor a little, a little and a very little) **22. 1.** Natural environment **22. 2.** Feed and Nutrition **22. 3.** Diseases and Injuries **22. 4.** Human Management **23. What do you think is the reason for being kind to farm animals?** (Strongly disagree; Disagree; Neither agree nor disagree; Agree; Strongly agree) **23. 1.** Contribution to humanity **23. 2.** Animals have spirituality **23. 3.** Animals are equal to humans **23. 4.** For better livestock products **23. 5.** Personal love and compassion for animals **23. 6.** Religious beliefs **23. 7.** Customs or family education **24. How important are the following conditions in animal care?** (Very important; Important; Neither important nor unimportant; Not important; Not important at all) **24. 1.** Sufficient and healthy diet **24. 2.** Clean and safe drinking water **24. 3.** Comfortable environment **24. 4.** Physical fitness **24. 5.** No diseases or injuries **24. 6.** Enough space **24. 7.** Natural instincts and behavioral expression **24. 8.** No fear **24. 9.** No pain **25. How important are the following conditions in animal care?** **25. 1.** Animal farms should have permits related to animal care **25. 2.** Procedures performed on animals such as ear tagging, spaying and neutering, and tail breaking are acceptable for management **25. 3.** Transportation time for live animals should be minimized **25. 4.** Farm animals should be provided with a comfortable and pleasant living environment. **25. 5.** Suffering animal products can be purchased if the quality is good enough **25. 6.** Suffering animal products can be purchased if the price is low enough **25. 7.** Animals should be unconscious until slaughtered **25. 8.** Animals should be completely dead before being cooked **25. 9.** Legislation to ensure animal protection is important **25. 10.** Animal protection organizations are important in ensuring that animals are adequately cared for
**Primary questions in bold.**
